# Molecular detection of a novel paramyxovirus in fruit bats from Indonesia

**DOI:** 10.1186/1743-422X-9-240

**Published:** 2012-10-19

**Authors:** Michihito Sasaki, Agus Setiyono, Ekowati Handharyani, Ibenu Rahmadani, Siswatiana Taha, Sri Adiani, Mawar Subangkit, Hirofumi Sawa, Ichiro Nakamura, Takashi Kimura

**Affiliations:** 1 Division of Molecular Pathobiology, Research Center for Zoonosis Control, Hokkaido University, Sapporo, Japan; 2 Division of Collaboration and Education, Research Center for Zoonosis Control, Hokkaido University, Sapporo, Japan; 3 Laboratory of Veterinary Pathology, Faculty of Veterinary Medicine, Bogor Agricultural University, Bogor, Indonesia; 4 Veterinary Investigation and Diagnostic Center, Bukittinggi, Indonesia; 5 Faculty of Agriculture, Gorontalo State University, Gorontalo, Indonesia; 6 Faculty of Animal Husbandry, Sam Ratulangi University, Manado, Indonesia

## Abstract

**Background:**

Fruit bats are known to harbor zoonotic paramyxoviruses including Nipah, Hendra, and Menangle viruses. The aim of this study was to detect the presence of paramyxovirus RNA in fruit bats from Indonesia.

**Methods:**

RNA samples were obtained from the spleens of 110 fruit bats collected from four locations in Indonesia. All samples were screened by semi-nested broad spectrum reverse transcription PCR targeting the paramyxovirus polymerase (*L*) genes.

**Results:**

Semi-nested reverse transcription PCR detected five previously unidentified paramyxoviruses from six fruit bats. Phylogenetic analysis showed that these virus sequences were related to henipavirus or rubulavirus.

**Conclusions:**

This study indicates the presence of novel paramyxoviruses among fruit bat populations in Indonesia.

## Background

The genus *Henipavirus* in the subfamily *Paramyxovirinae*, family *Paramyxoviridae*, contains two highly pathogenic viruses, i.e., Hendra virus and Nipah virus. Hendra virus causes fatal pneumonia and encephalitis in horses and humans. The first case was identified in 1994 and Hendra virus disease still continues to arise sporadically in Australia [1,2]. Nipah virus also causes acute encephalitis and respiratory symptoms in animals and humans, with a high mortality rate. Outbreaks of Nipah virus have occurred in Malaysia, Singapore, Bangladesh, and India [1,2]. Henipaviruses have been isolated from fruit bats including *Pteropus vampyrus*[3], *Pteropus hypomelanus*[4], *Pteropus lylei*[5], *Pteropus poliocephalus,* and *Pteropus alecto*[6], which are considered to be their natural reservoirs. Epidemiological studies demonstrate that Hendra and/or Nipah virus-seropositive fruit bats are widely distributed throughout Asian countries [7-11]. No human cases of henipavirus infection have been reported in Indonesia, although *Pteropus vampyrus* that are seropositive for both Nipah virus and Hendra virus are distributed nationwide [12,13]. These findings indicate the presence of henipavirus or henipa-like viruses in Indonesian fruit bats, suggesting the need for further epidemiological investigations.

Menangle virus, belonging to the genus *Rubulavirus* of the *Paramyxoviridae* family, has been identified in pteropus bats from Australia [14]. Menangle virus is a zoonotic paramyxovirus that causes febrile illness with rash in humans [15]. Tioman virus, belonging to the genus *Rubulavirus*, has also been isolated from *Pteropus hypomelanus* on the island of Tioman, Malaysia [16]. Although Tioman virus showed antigenic cross-reactivity to Menangle virus, the pathogenicity of Tioman virus remains unclear. There have been no reports of rubulavirus infections in the Indonesian fruit bat population.

The current study used molecular sequencing and phylogenetic analyses to identify RNA sequence from potential paramyxoviruses in fruit bats from Indonesia.

## Results

**Figure 1:**
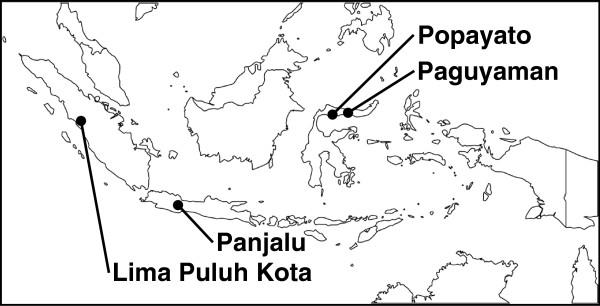
Map of the sampling locations in Indonesia.

A total of 110 fruit bats belonging to four different species were sampled from four locations in Indonesia (Figure 1). *Pteropus vampyrus* was captured in Panjalu District (n = 26) and Lima Puluh Kota District (n = 20). Other pteropus bats captured in Popayato District (n = 4) and Paguyaman District (n = 25) were considered to be closely related to *Pteropus hypomelanus*, based on the shared nucleotide sequence identity of their 16S rRNA (96%) and cytochrome *b* (cyt *b*) (95%) with corresponding sequences from *Pteropus hypomelanus* (GenBank/EMBL/DDBJ entry AF069537 and AB062472)*. Acerodon celebensis* was captured in Paguyaman District (n = 18). Dobsonia bats that were captured in Paguyaman District (n = 17) had high sequence similarity with 16S rRNA (96%) and cyt *b* (94%) from *Dobsonia moluccensis* (JN398196 and FJ218484). Information on the samples is summarized in Table 1.

**Table 1:** Sample information and result of the semi-nested RT-PCR

**Species**	**Location collected**	**Year collected**	**Nested RT-PCR**	
			**Number of analyzed**	**Number of positive**
*Pteropus vampyrus*	Panjalu district	2010	26	1
	Lima Puluh Kota district	2011	20	0
*Pteropus* sp.*	Popayato district	2011	4	0
	Paguyaman district	2011	23	4
		2012	2	0
*Acerodon celebensis*	Paguyaman district	2012	18	1
*Dobsonia* sp.**	Paguyaman district	2012	17	0

*Genetically closely related to *Pteropus hypomelanus*.

**Genetically closely related to *Dobsonia moluccensis*.

RNA samples from each fruit bat spleen were screened using semi-nested broad spectrum reverse transcription PCR (RT-PCR), as described previously [17]. The primers were designed based on a conserved sequence within the RNA polymerase large (*L*) gene of the *Paramyxovirinae* subfamily, which includes *Avulavirus*, *Rubulavirus*, *Respirovirus*, *Morbillivirus*, and *Henipavirus*[17]. Semi-nested RT-PCR was positive for 1/26 (4%) *Pteropus vampyrus* specimens captured in Panjalu District. The size of PCR product detected in the positive sample (sample number IFBPV01/2010) was 584 bp, and the amplified viral sequence excluding the primer-derived sequences (530 bp) was deposited in GenBank (accession number AB691542). Positive results with amplification of the 530 bp viral sequence (excluding the primer-derived sequences) were also obtained for 4/25 (16%) *Pteropus* sp. captured in Paguyaman District, i.e., IFBPV25/2011 (AB691543), IFBPV32/2011 (AB691544), IFBPV39/2011 (AB691545), and IFBPV46/2011 (AB691546), and for 1/18 (6%) *Acerodon celebensis* specimens captured in Paguyaman District, i.e., IFBPV32/2012 (AB710472). No positive results were obtained for the 20 *Pteropus vampyrus* captured in Lima Puluh Kota District, the four *Pteropus* sp. captured in Popayato District, or the 17 *Dobsonia* sp. captured in Paguyaman District (Table 1).

BLAST search showed that all six amplicons shared less than 65% nucleotide identity with homologous fragments of paramyxovirus sequences previously deposited in GenBank. Deduced pairwise amino acid identities were then calculated to compare the homologous region with known paramyxovirus L proteins (Table 2). IFBPV32/2011 shared 98% nucleotide identity and 100% amino acid identity with IFBPV39/2011, suggesting that they belonged to the same strain. IFBPV01/2010, IFBPV32/2011, IFBPV39/2011, and IFBPV46/2011 were most closely related to Nipah virus of all the known paramyxoviruses. IFBPV25/2011 shared 72% amino acid sequence identity with Tuhoko virus 2, which was isolated from *Rousettus leschenaulti* in China [18]. IFBPV32/2012 shared 78% amino acid sequence identity with Tioman virus.

**Table 2:** **Pairwise amino acid identities of predicted *L *gene products compared with known paramyxoviruses**

**Genus**	**Species**	**Percentage of amino acid sequence identity**				
		**IFBPV01/2010**	**IFBPV25/2011**	**IFBPV32/2011, IFBPV39/2011**	**IFBPV46/2011**	**IFBPV32/2012**
	IFBPV01/2010		37	90	66	35
	IFBPV25/2011	37		36	38	70
	IFBPV32/2011, IFBPV39/2011	90	36		65	36
	IFBPV46/2011	66	38	65		38
	IFBPV32/2012	35	70	36	38	
*Avulavirus*	Avian paramyxovirus 3	29	36	30	34	35
	Newcastle disease virus	34	37	34	36	40
*Rubulavirus*	Mapuera virus	41	65	40	40	64
	Menangle virus	34	65	33	37	77
	Mumps virus	38	65	36	40	68
	Tioman virus	34	69	34	37	78
	Tuhoko virus 2	38	72	36	38	72
*Respirovirus*	Human parainfluenza virus 1	51	35	49	52	34
	Human parainfluenza virus 3	51	37	51	52	35
	Sendai virus	51	35	49	51	34
*Morbillivirus*	Canine distemper virus	57	37	56	55	34
	Measles virus	58	37	57	56	37
	Rinderpest virus	57	36	57	56	38
*Henipavirus*	Hendra Virus	67	36	66	69	35
	Nipah Virus	70	36	69	70	35
Unclassified	J-virus	61	38	62	60	38

**Figure 2:**
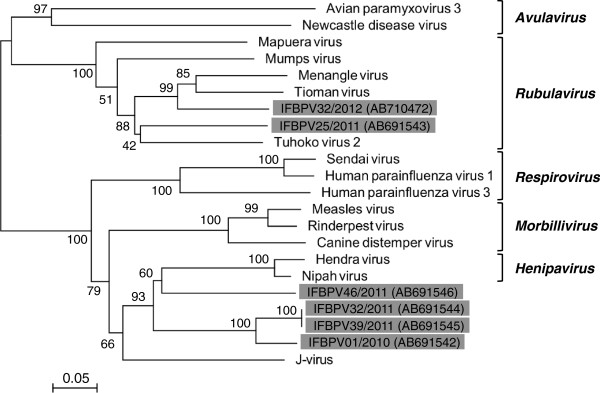
**Phylogenetic analysis of amino acid sequences derived from partial *L *gene fragments.** The phylogenetic tree was constructed based on the 176 amino acid sequences deduced from the partial *L* gene fragments identified in the present study (gray shade) and homologous sequences from known paramyxoviruses. The bootstrap values obtained after 1000 replicates are indicated at each branch. Scale bars indicate amino acid substitutions per site.

A phylogenetic analysis was performed based on the deduced amino acid sequences (176 amino acids) from the six nucleotide sequences obtained (Figure 2). The phylogenetic tree showed that IFBPV01/2010, IFBPV32/2011, IFBPV39/2011, and IFBPV46/2011 formed three distinct branches that were closely related to the genus *Henipavirus*. IFBPV25/2011 and IFBPV32/2012 were most closely related to the genus *Rubulavirus*.

We also amplified other regions of *L* gene by using different degenerate primer sets for *MorbillivirusRespirovirusHenipavirus* subgroup or *RubulavirusAvulavirus* subgroup [17]. The partial viral sequences measuring 439 bp (excluding the primer-derived sequences) was obtained from four henipavirus-like RNA-positive samples, i.e., IFBPV01/2010 (AB748559), IFBPV32/2011 (AB748560), IFBPV39/2011 (AB748560), and IFBPV46/2011 (AB748561). A phylogenetic analysis on the deduced amino acid sequences showed that IFBPV01/2010, IFBPV32/2011, IFBPV39/2011 and IFBPV46/2011 were related to the genus *Henipavirus* (Additional file 1). The viral sequence measuring 169 bp (excluding the primer-derived sequences) was obtained from two rubulavirus-like RNA-positive samples, i.e., IFBPV25/2011 (AB691543) and IFBPV32/2012 (AB710472). Phylogenetic analysis performed on the deduced amino acid sequences showed that IFBPV25/2011 and IFBPV32/2012 were related to the genus *Rubulavirus* (Additional file 2).

**Figure 3:**
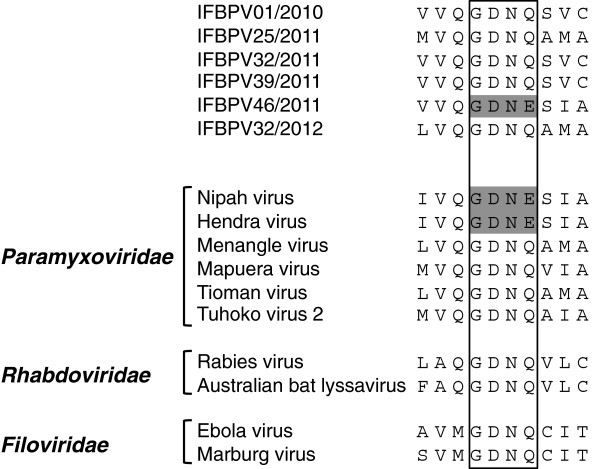
**Amino acid alignment of the GDNQ/GDNE motif.** An amino acid alignment of the GDNQ/GDNE motif was constructed based on the sequences of nonsegmented negative-stranded RNA virus RNA polymerase, including paramyxovirus L protein. The conserved GDNQ/GDNE sequences are boxed while GDNE is also shaded in gray.

The amino acid sequence GDNQ is highly conserved in the viral RNA polymerase of non-segmented negative-stranded RNA viruses and it is responsible for polymerase activity [19,20]. However, this motif is replaced by GDNE in the L protein of *Henipavirus*[20,21]. The region encoding the GDNQ/GDNE motif was amplified by RT-PCR to determine whether the putative henipavirus-like nucleotide sequences contained the characteristic GDNE motif in the L protein. The deduced amino acid sequence comparison showed that only IFBPV46/2011 encoded the GDNE motif, among the six samples obtained (Figure 3).

Virus isolation was attempted using African green monkey kidney (Vero) and rabbit kidney (RK13) cells because these cell lines are used for the isolation or propagation of various paramyxoviruses [3,4,6,16,22]. After serial passages, an RT-PCR assay detected no paramyxovirus RNA in the culture supernatants (data not shown).

## Discussion

Four henipavirus-like and two rubulavirus-like nucleic acid sequences were detected in fruit bats from Indonesia. The phylogenetic analysis showed that these novel viral sequences possessed considerable sequence divergence, suggesting that a variety of paramyxoviruses are circulating in the Indonesian fruit bat population.

In addition to fruit bats of the genus *Pteropus*, partial paramyxovirus sequences were identified from fruit bats of the genus *Eidolon*, *Rousettus* and *Epomophorus*[18,23,24]. To our knowledge, this is the first study that has detected paramyxovirus RNA from fruit bats of the genus *Acerodon*. This finding broadens the number of megabat genera which are associated with paramyxoviruses.

Tuhoko virus, Tioman virus, Menangle virus, and Mapuera virus have been identified as fruit bat-associated rubulaviruses. Menangle virus causes central nervous system degeneration in pigs and it also infects humans [14,15]. Therefore, it would be useful to investigate infections of humans or domestic animals with the novel rubula-like viruses detected in this study.

## Conclusions

This study identified unique paramyxovirus sequence from three species of fruit bats (*Pteropus vampyrus*, *Pteropus hypomelanus* and *Acerodon celebensis*), potentially representing three new henipaviruses and two new rubulaviruses. To the best of our knowledge, this is the first study to identify viral genome sequence from potential paramyxoviruses in the tissues of fruit bats from Indonesia. Local people consume bat meat in Indonesia, so further epidemiological and experimental studies are needed to determine the risk of fruit bat-associated paramyxovirus infection of humans in Indonesia.

## Methods

### Animal samples and RNA extraction

Fruit bats were captured in: Panjalu District, West Java Province during February, 2010 (n = 26); Lima Puluh Kota District, West Sumatra Province during February, 2011 (n = 20); Popayato District, Gorontalo Province during February, 2011 (n = 4); and Paguyaman District, Gorontalo Province during February, 2011 and February, 2012 (n = 23 and n = 37, respectively). All animal research was performed in accordance with the ethical guidelines of the Animal Care and Use Committee of Veterinary Teaching Hospital, Bogor Agricultural University. All of the spleen tissues from fruit bats were divided into two samples. Samples for RNA extraction were stored in RNAlater (Life Technologies, Carlsbad, CA), followed by RNA extraction with TRIzol reagent (Life Technologies) according to the manufacturer’s instructions. Samples for virus isolation were frozen at −80°C. These samples were exported with the permission of the Directorate General of Livestock and Animal Health Services, Ministry of Agriculture, Republic of Indonesia.

### Species identification

Fruit bats were identified based on morphological characters and nucleotide sequence analysis of their mitochondrial 16S rRNA using the primer set 12l-f (5^′^-AGAGGAGAYAAGTCGTAMCAAG-3^′^) [25] and 16q-r (5^′^-GTTTGCCGAGTTCCTTTTAC-3^′^) [25], and mitochondrial cyt *b* using the primer set Ptecytb-26 (5^′^-TTGTATTTCAACTACARGAAC-3^′^) designed in this study and H15149p (5^′^-CTGCAGCCCCTCAGAATGATATTTGTCCTC-3^′^) [26].

### RT-PCR

RNA samples were screened for paramyxoviruses using semi-nested RT-PCR, as described previously [17]. The primer annealing temperature of the PCR programs was modified to 48°C. The degenerate primers used for amplification of the *L* gene of the *Paramyxovirinae* subfamily were as follows: for one-step RT-PCR, PAR-F1 and PAR-R; for semi-nested PCR, PAR-F2 and PAR-R [17]. The upstream region of *L* gene of the *Respirovirus-MorbillivirusHenipavirus* subgroup or *AvulavirusRubulavirus* subgroup were amplified by using the following degenerate primer sets: RES-MOR-HEN F1, RES-MOR-HEN F2 and RES-MOR-HEN R, or AVU-RUB F1, AVU-RUB F2 and AVU-RUB R, respectively [17]. PCR products were electrophoresed on 1.6% agarose gel and purified with a QIAquick Gel Extraction Kit (Qiagen, Valencia, CA). Direct cycle sequence reactions were performed in both directions using a BigDye Terminator v3.1 Cycle Sequencing Kit (Life Technologies) and analyzed with an ABI Prism 3130 genetic analyzer (Life Technologies).

The nucleotide sequence encoding the GDNQ/GDNE motif from each sample was amplified using a SuperScript III One-Step RT-PCR System with Platinum Taq DNA Polymerase (Life Technologies). The inner primer PAR-F2 used in the semi-nested PCR was designed for a nucleotide sequence encoding this GDNQ/GDNE motif, so one-step RT-PCR was performed with the outer forward primer PAR-F1 and reverse primers specifically for each sample.

### Phylogenetic analysis

The obtained nucleotide sequences and the deduced amino acid sequences were compared with those of known paramyxoviruses. Nucleotide and amino acid identity values were calculated using GENETYX software ver. 10 (GENETYX, Tokyo, Japan). Multiple sequence alignments were constructed based on the amino acid sequences deduced from the six nucleotide sequences obtained using the MEGA5 program [27]. Phylogenetic analysis was performed using the neighbor-joining method with 1000 bootstrap replicates [28,29].

### Viral isolation

Virus isolation was attempted in the biosafety level (BSL)-3 facility at the Research Center for Zoonosis Control, Hokkaido University. Frozen spleen tissues were homogenized (10%, wt/vol) in MEM containing penicillin (100 units/ml), streptomycin (100 μg/ml), and fungizone (2.5 μg/ml) (all obtained from Life Technologies). The homogenates were clarified by centrifugation at 1000 × *g* for 5 min and inoculated onto Vero cells and RK13 cells for 2 h at 37°C. Cells were washed with phosphate-buffered saline (−) and cultured with MEM containing 2% fatal bovine serum, penicillin (100 units/ml), streptomycin (100 μg/ml) and fungizone (2.5 μg/ml). All cells were subcultured every 5 or 6 days. After three serial passages, RNA samples were prepared from each culture supernatant using a High Pure Viral RNA Kit (Roche Diagnostics, Mannheim, Germany) and analyzed by semi-nested RT-PCR, as described above.

### Accession number

The amplified nucleotide sequences reported in this study have been deposited in the GenBank nucleotide database under accession numbers AB691542 to AB691546, AB748559 to AB748561 and AB710472. AB691543 and AB710472 are composed of three overlapping sequences that were amplified using three primer sets; PAR-F2 and PAR-R, AVU-RUB F2 and AVU-RUB R, and the primers for the amplification of GDNQ/GDNE motif.

The GenBank/EMBL/DDBJ accession numbers of the amino acid sequences used in this study were ACB46872 (Avian paramyxovirus 3), NP_071471 (Newcastle disease virus), NP_054714 (Mumps virus), YP_001249278 (Mapuera virus), YP_415514 (Menangle virus), NP_665871 (Tioman virus), ADI80722 (Tuhoko virus 2), NP_056879 (Sendai virus), NP_604442 (Human parainfluenza virus 1), NP_067153 (Human parainfluenza virus 3), AAA75501 (Measles virus), YP_087126 (Rinderpest virus), NP_047207 (Canine distemper virus), NP_047113 (Hendra virus), NP_112028 (Nipah virus), and AAX86035 (J-virus), NP_056797 (Rabies virus), NP_478343 (Australian bat lyssavirus), NP_066251 (Ebola virus), and ACT79225 (Marburg virus).

## Competing interests

The authors declare that they have no competing interests.

## Authors’ contributions

M. Sasaki, A. Setiyono, E. Handharyani, and T. Kimura designed research; M. Sasaki, A. Setiyono, E. Handharyani, S. Adiani, I. Rahmadani, S. Taha, M. Subangkit, I. Nakamura, and T. Kimura performed research; M. Sasaki, A. Setiyono, E. Handharyani, H. Sawa and T. Kimura analyzed the data; M. Sasaki, H. Sawa and T. Kimura wrote the manuscript. All authors read and approved the final manuscript.

## Supplementary Material

Additional file 1 **Phylogenetic analysis of amino acid sequences derived from partial *L *gene fragments.** Click here for file

Additional file 2 **Phylogenetic analysis of amino acid sequences derived from partial *L *gene fragments.** Click here for file
