# Supplementary material for: Molecular detection of a novel paramyxovirus in fruit bats from Indonesia
Source: Virol J. 2012 Oct 19;9:240. doi: 10.1186/1743-422X-9-240 (PMC3499202; doi:10.1186/1743-422X-9-240)
Supplement: Additional file 2 — Phylogenetic analysis of amino acid sequences derived from partial L gene fragments. [file 1743-422X-9-240-S2.pdf]

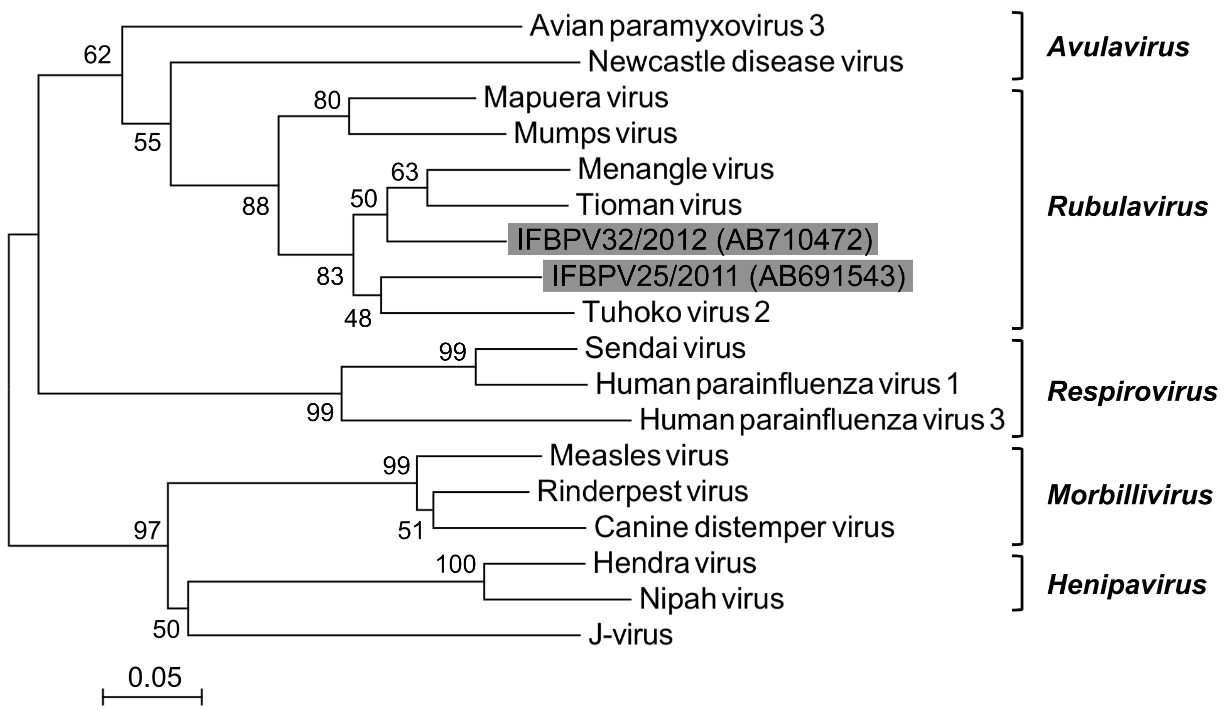


**Supporting Figure 2 - Phylogenetic analysis of amino acid sequences derived from partial *L* gene fragments**

A 169 bp segment of *L* gene of IFBPV25/2011 and IFBPV32/2012 was obtained by using the degenerate primer set for the *Avulavirus*-*Rubulavirus* subgroup. Phylogenetic tree was constructed from the deduced amino acid sequence (56 amino acids) and homologous sequences from known paramyxoviruses. The bootstrap values obtained after 1000 replicates are indicated at each branch. Scale bars indicate amino acid substitutions per site.
